# EHMT2 inhibitor BIX-01294 induces endoplasmic reticulum stress mediated apoptosis and autophagy in diffuse large B-cell lymphoma cells

**DOI:** 10.7150/jca.48310

**Published:** 2021-01-01

**Authors:** Linyan Xu, Xiang Gao, Pu Yang, Wei Sang, Jun Jiao, Mingshan Niu, Mengdi Liu, Yuanyuan Qin, Dongmei Yan, Xuguang Song, Cai Sun, Yu Tian, Feng Zhu, Xiaoshen Sun, Lingyu Zeng, Zhenyu Li, Kailin Xu

**Affiliations:** 1 Blood Diseases Institute, Xuzhou Medical University, Xuzhou, Jiangsu, China.; 2 Department of Hematology, the Affiliated Hospital of Xuzhou Medical University, Xuzhou, Jiangsu, China.; 3 Key Laboratory of Bone Marrow Stem Cell, Xuzhou, Jiangsu, China.; 4 Department of Hematology, Luoyang Central Hospital Affiliated to Zhengzhou University, Luoyang, Henan, China.

**Keywords:** apoptosis, autophagy, BIX-01294, diffuse large B cell lymphoma, endoplasmic reticulum stress

## Abstract

Despite advancement in the treatment of diffuse large B-cell lymphoma (DLBCL), many patients tend to relapse or become refractory after initial therapy. Therefore, it is essential to identify novel therapeutic targets and drugs, understand the molecular pathogenesis mechanism of DLBCL, and find ways to prevent and treat relapsed or refractory DLBCL. BIX-01294 is a small molecule compound that specifically inhibits EHMT2 activity. In this study, we demonstrate that BIX-01294 triggered the inhibition of human DLBCL cell proliferation, lead to G_1_ phase arrest via increasing *P21* level and reducing *cyclin E* level. BIX-01294 also induced apoptosis via endogenous and exogenous apoptotic pathways. Moreover, BIX-01294 triggered autophagy and activated ER stress in human DLBCL cells. Furthermore, we showed that both key components of ER stress, ATF3, and ATF4, are required for BIX-01294-induced apoptosis and autophagy. Hence, this study provides new evidence that EHMT2 may be a new therapeutic target, and BIX-01294 may be a potential therapeutic drug for treating DLBCL.

## Introduction

Diffuse large B-cell lymphoma (DLBCL) is the most common type of lymphoma and represents 30-40% of non-Hodgkin lymphoma (NHL) cases worldwide. At present, the standard therapy R-CHOP (rituximab, cyclophosphamide, doxorubicin, vincristine and prednisone) regimen may lead to complete remission. However, 30-40% of patients are refractory to therapy or relapse after treatment indicating standard cytotoxic therapy has its limitations 1-4. Factors that influence the cure of DLBCL include patient age, molecular subtypes, gene expression, and others 5. Therefore, it is essential to identify novel therapeutic targets and drugs, understand the molecular pathogenesis mechanism of DLBCL and find ways to prevent and treat relapsed/refractory DLBCL.

Epigenetic regulation plays an important role in gene expression and also influences tumor progression in many types of cancers, of which histone methylation is a vital posttranslational modification 6-8. Histone lysine N-methyltransferase EHMT2 (G9a) is a methyltransferase that is mainly responsible for catalyzing mono- and di-methylation of histone H3 lysine 9 (H3K9) in euchromatic regions, and inhibits transcription 9. Recently, abnormal expression of EHMT2 has been discovered in various types of malignant cells, including acute myeloid leukemia, bladder cancer, lung cancer, squamous cell carcinoma, ovarian cancer, etc. 10-12. Cancers with elevated expression levels of EHMT2 often show lower expression of some important tumor suppressor genes and epithelial-to-mesenchymal transition (EMT) related genes, so its higher expression may contribute to poor prognosis 13-16. Studies have proven that inhibiting EHMT2 expression suppressed cell growth and inhibited cancer cell migration and invasion 17. So EHMT2 is an important methyltransferase and may be an excellent target for anticancer therapy.

BIX-01294 is a small molecule compound that specifically inhibits EHMT2 activity and induces demethylation of H3K9 18. Studies have shown that BIX-01294 inhibited cell proliferation and induced apoptosis in many types of cancers including T lymphoblastic leukemia cells, bladder cancer, glioma tumor, renal cancer, and others 10, 19-21. However, the role of BIX-01294 and the involvement of EHMT2 in DLBCL is not well understood.

In this study, we investigated the effect of BIX-01294 on the proliferation and cell cycle of DLBCL cells, confirmed BIX-01294 induced apoptosis both in extrinsic and intrinsic pathways. Meanwhile, we explored the possible molecular mechanism of this anti-tumor effect.

## Materials and Methods

### Cell culture

The human DLBCL cell lines U2932, SUDHL2, OCI-LY10, WSU-DLCL2, and DB were obtained from the American Type Culture Collection (Manassas, VA, USA). Cells were grown in IMDM medium and cultured with 10% fetal bovine serum at 37 °C in a humidified atmosphere consisting of 5% CO_2_. Peripheral blood samples were obtained from two healthy donors. Peripheral blood mononuclear cells (PBMC) were separated using Lymphoprep™ (STEMCELL Technologies, Canada) by centrifugation and cultured with 10% fetal bovine serum.

### Antibodies and reagents

BIX-01294 in powder form was purchased from Sigma-Aldrich (Saint Louis, Missouri, USA). EHMT2, DR4, DR5, caspase 3, PARP, Bax, Mcl-1, GRP78, ATF3, CHOP, LC3B, ATF4, β-actin and GAPDH antibodies were purchased from Cell Signaling Technology (Danvers, MA, USA). The c-FLIP antibody was purchased from EZNO Life Sciences (Farmington, NY, USA).

### Cell viability assay

Human DLBCL cell lines were seeded in 96-well plates with 2×10^4^ cells per well and treated with BIX-01294 with the indicated concentrations for the indicated times. Then cell viability was measured using Cell Counting Kit-8 (CCK-8) according to the manufacturer's instructions (Dojindo Laboratories, Kumamoto, Japan).

### Western blot

Human DLBCL cell lines were seeded in 6-well plates with 2×10^6^ cells per well and treated with BIX-01294 with the indicated concentrations for the indicated times. Cells were harvested and lysed with cell lysis buffer containing protease inhibitor. BCA assay was used to detect protein concentrations and 50 µg protein was used for sample preparation. The sample was electrophoresed and transferred to PVDF membranes and then blocked with 5% skim milk and incubated with primary antibodies overnight. Next day, the membranes were washed with PBST and incubated with HRP-conjugated secondary antibodies, and protein bands were subsequently captured. The relative density of bands was quantified using Image J software.

### Apoptosis assay

Human DLBCL cell lines were seeded in 6-well plates with 2×10^6^ cells per well and treated with BIX-01294 with the indicated concentrations for indicated times. Then cells were harvested, and apoptosis was detected using Annexin V-PE/7-AAD Apoptosis Detection Kit (BD Biosciences, San Jose, CA, USA) following the manufacture's protocol.

### Cell cycle analysis

Human DLBCL cell lines were seeded in 6-well plates with 2×10^6^ cells per well and treated with indicated concentrations of BIX-01294 for 36 h. Cells were harvested and fixed with 70% ice-cold ethanol at 20 °C overnight. Then cells were washed with PBS and resuspended with 0.25 mL staining solution (50 µg/mL RNase A and 50 µg/mL propidium iodide) for 30 min in the dark, followed by cytometric analysis using FACS Calibur system (BD Biosciences, Franklin Lakes, NJ, USA) and analyzed using ModFit LT 3.3 software (Verity Software House Inc., Topsham, ME, USA).

### RNA extraction and real-time qPCR

The cells were harvested, and RNA was extracted with TRIzol^™^ reagent according to the manufacturer's instruction. The concentration was measured at 260 nm and then reverse-transcripted to cDNA (Roche, Basel, Switzerland). Q-PCR was performed with SYBR^®^ Green Supermix (Roche) using LightCycler480 according to the manufacturer's instruction. The primer sequences are shown in Table 1. The PCR reaction conditions were as follows: 94 °C for 2 min, followed by 35 cycles of 94 °C for 30 s, 60 °C for 30 s, 72 °C for 35 s, and a final extension at 72 °C for 2 min. The 2^-ΔΔCT^ method was used to calculate the relative mRNA expression of target genes.

### ShRNA design and generation of lentiviruses and construction of stable cell lines

The ATF4 small-hairpin RNA (shRNA) primer sequences were designed as 5′-GCCTAGGTCTCTTAGATGATT-3′ (1), 5′-GCCAAGCACTTCAAACCTCAT-3′ (2). ATF3 shRNA sequences were designed as 5′-GCTGAACTGAAGGCTCAGATT -3′ (1), 5′-GCACCTCTGCCACCGGATG-3′ (2). The shRNA sequences were constructed into a pLVshRNA-eGFP vector. HEK-293T cells were transfected with 10 µg desired plasmid, 7.5 µg of the packaging plasmid psPAX2 and 2.5 µg of the envelope plasmid pMD2.G using Lipofectamine 2000 (Invitrogen, Carlsbad, CA, USA) according to the manufacturer's instruction. After 12 h, the culture media was exchanged, and the lentivirus supernatant was collected at 48 h and 72 h for concentration and stored at -80 °C. U2932, WSU-DLCL2, and SUDHL2 cell lines were transduced with lentivirus, screened the stable knockdown cell lines by puromycin, and identified using flow cytometry and western blotting.

### Statistical analysis

Data were shown as means ± standard deviation (SD). Two-tailed unpaired Student's *t*-test was used to analyze the statistical significance between two treatment groups. One-way ANOVAs was used to analyze the statistical significance of multiple treatment groups. Statistical significance was established at *P<0.05, **P<0.01, ***P<0.001. All statistical analyses were performed using the GraphPad Prism software ver. 6.0 (GraphPad Software Inc., La Jolla, CA, USA).

## Results

### High EHMT2 expression predicts poor survival in DLBCL patients and BIX-01294 inhibits EHMT2 expression in human DLBCL cells

**Figure 1:**
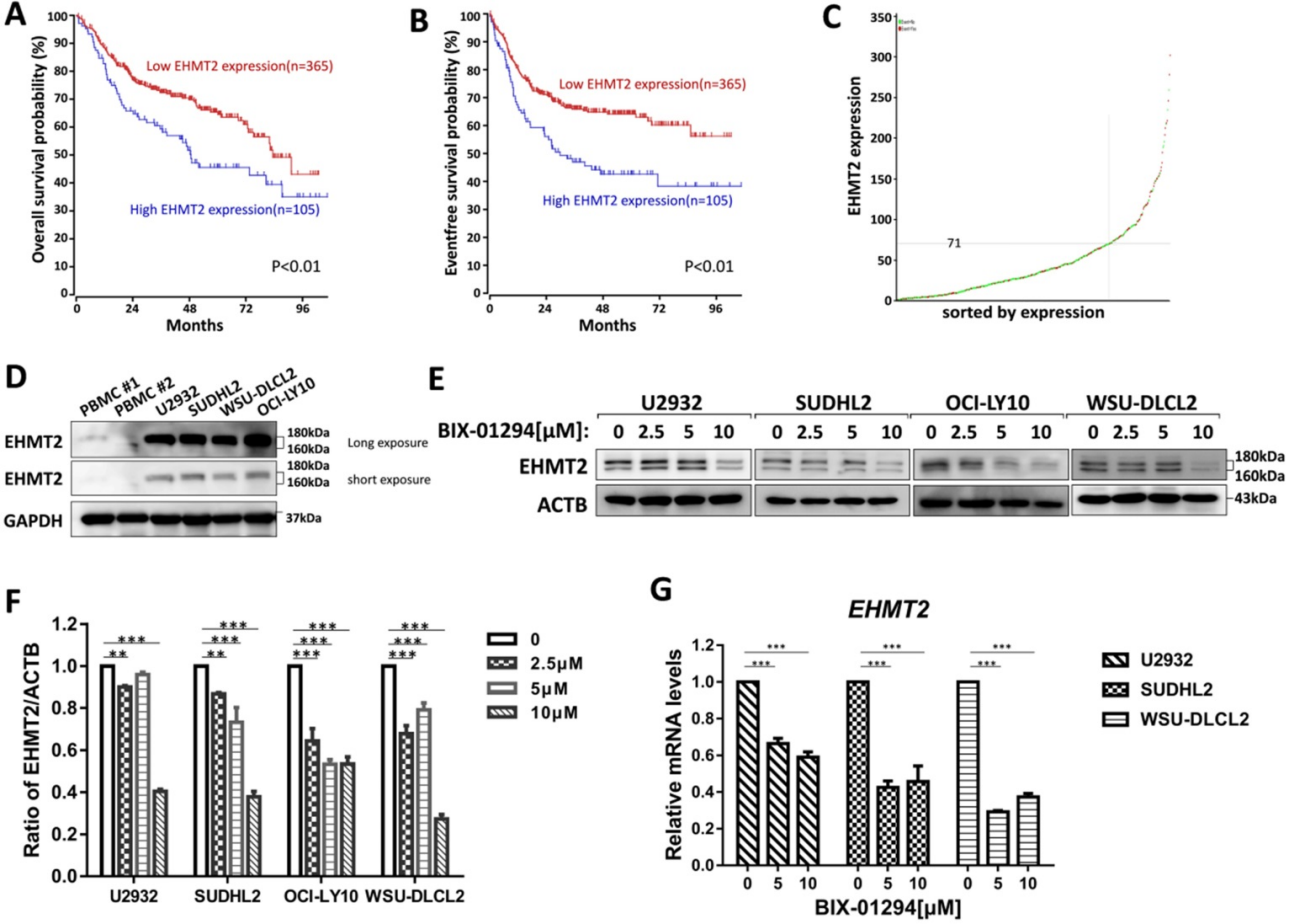
** BIX-01294 inhibits EHMT2 expression in human DLBCL cells. A,** Kaplan-Meier analysis of OS from R2 genomics database. EHMT2 was high expression in 105 out of 470 cases of DLBCL and with poor patient survival. **B,** Kaplan-Meier analysis of EFS from R2 genomics database. **C,** The expression level of EHMT2 gene from 470 cases of DLBCL patients. **D,** U2932, SUDHL2, WSU-DLCL2, and OCI-LY10 cells and human PBMC were harvested and subjected to western blot analysis using EHMT2 antibody. **E,** U2932, SUDHL2, OCI-LY10, and WSU-DLCL2 cells were incubated with the indicated concentrations (0, 2.5 µm, 5 µm, 10 µm) of BIX-01294 for 48 h. Then whole cells were harvested and subjected to western blot analysis using EHMT2 antibody. **F,** Gray value analysis of corresponding protein of E. **G,** U2932, SUDHL2, and WSU-DLCL2 cells were incubated with the indicated concentrations of BIX-01294 for 36 h. The expression of *EHMT2* mRNA was assessed by real-time PCR. Error bars mean ± SD. **P < 0.01; ***P < 0.001.

To investigate the prognostic significance of the EHMT2 expression, we analyzed the microarray-based gene expression and clinical outcome data from the R2 genomics database. The Kaplan-Meier analysis was conducted online, and auto scan mode was used to determine the cutoff values for separating the high and low expression groups. As shown in Fig. 1A-1C, patients with high EHMT2 expression had a worse Overall survival (OS) and Event-free survival (EFS) compared with patients with low EHMT2 expression (*P<0.01*), indicating that high expression of EHMT2 had a prognostic value for predicting EFS and OS. Next, we assessed the expression of EHMT2 in DLBCL cells and peripheral blood mononuclear cells (PBMC) from healthy donors, and found PBMC minimally expressed EHMT2, while DLBCL cells highly expressed EHMT2 (Fig. 1D). Several DLBCL cells were treated with BIX-01294 at different concentrations for 48 h, and western blot showed that BIX-01294 inhibited EHMT2 expression (Fig. 1E and 1F). Using qPCR assay, the mRNA levels of EHMT2 were down-regulated in U2932, SUDHL2, and WSU-DLCL2 cells (Fig. 1G).

### BIX-01294 inhibits DLBCL proliferation and induces G_1_ phase arrest in human DLBCL cells

**Figure 2:**
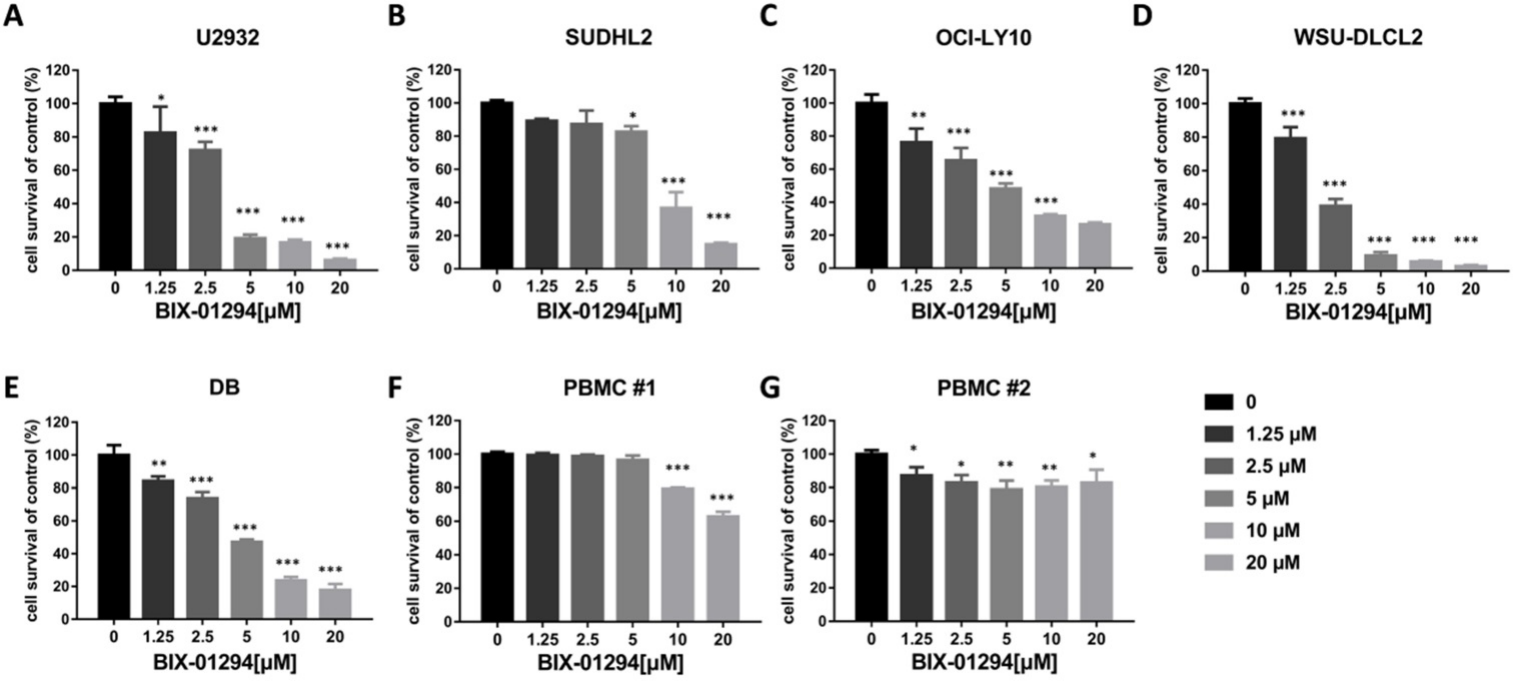
** BIX-01294 inhibits human DLBCL cells proliferation. A-G.** U2932 (A), SUDHL2 (B), OCI-LY10 (C), WSU-DLCL2 (D), DB (E) and human PBMC (F and G) were incubated with the indicated concentrations of BIX-01294 for 48 h, then CCK-8 assay was performed to detect the viability. Error bars, mean ± SD. Compare with the control group, *P < 0.05; **P < 0.01; ***P < 0.001.

**Figure 3:**
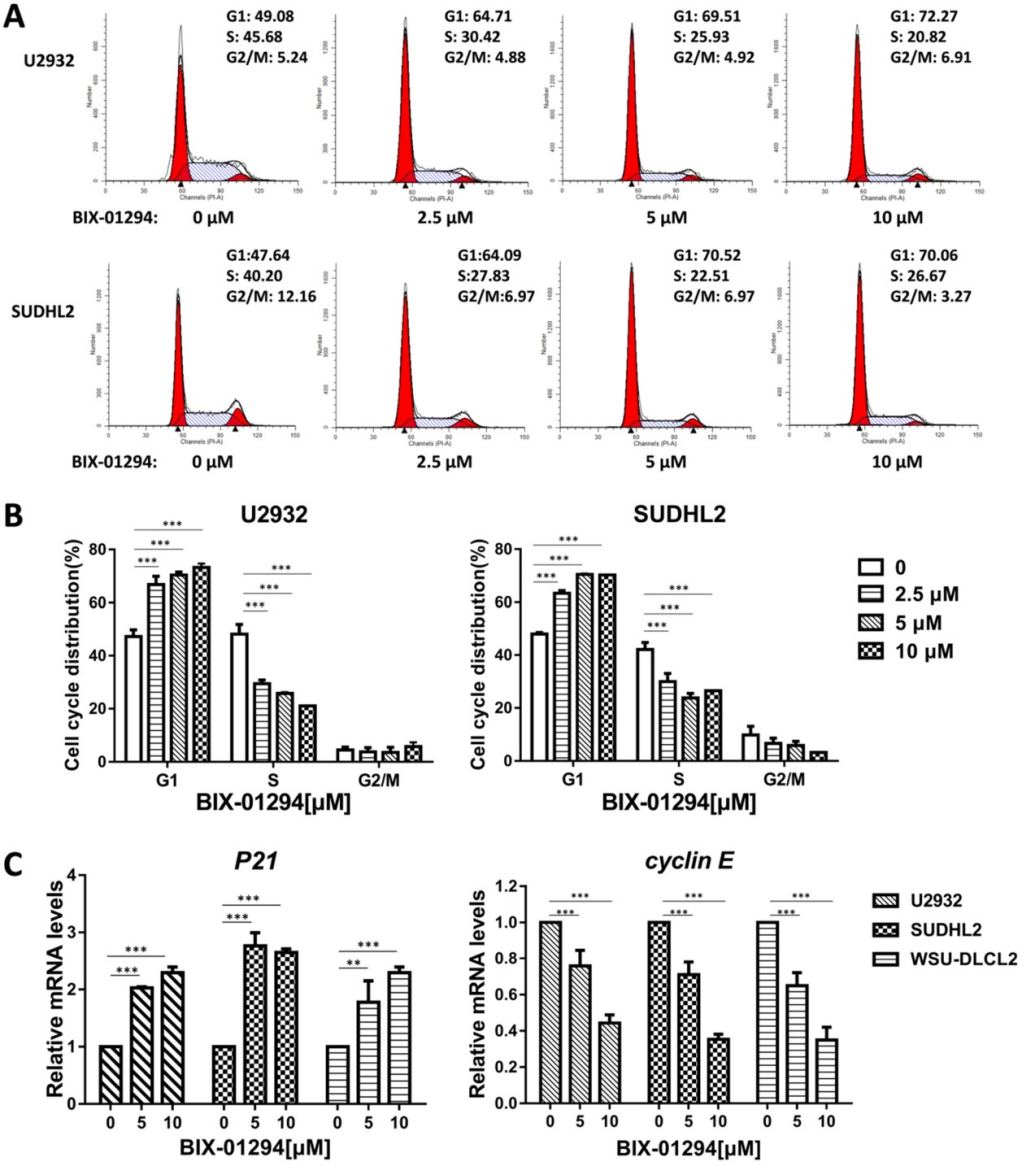
** BIX-01294 induces G_1_ phase arrest in human DLBCL cells. A,** U2932 and SUDHL2 cells were incubated with the indicated concentrations (0, 2.5 µm, 5 µm, 10 µm) of BIX-01294 for 36 h, and then the cells were harvested and prepared for cell cycle analysis. **B,** Percentages of subpopulation of cells at different cell cycle phases based on three independent experiments. **C,** U2932, SUDHL2, and WSU-DLCL2 cells were incubated with the indicated concentrations of BIX-01294 for 36 h. The expression of *P21* and *cyclin E* mRNA was assessed by real-time PCR. Error bars, mean ± SD. **P < 0.01; ***P < 0.001.

To determine the effect of BIX-01294 on DLBCL cells growth status, CCK-8 assay was performed to detect the viability. As shown in Fig. 2A-E, BIX-01294 exhibited notable proliferation suppression in a dose-dependent manner in five DLBCL cells, no matter the ABC type or GCB type cells. At the same time, we examined the inhibitory effect of BIX-01294 on PBMC, and there was a slight inhibition effect compared to that in DLBCL cells (Fig. 2F and 2G), indicating BIX-01294 exerted a relatively specific inhibition effect for tumor cells. To further study the underlying mechanism that BIX-01294 inhibited DLBCL cells proliferation, flow cytometry analysis was carried out to investigate the cell cycle distribution when treated with BIX-01294 (Fig. 3A and 3B). The cell population in G_1_ phase was increased both in U2932 and SUDHL2 cells, accompanied with the population in S phase decreasing from 45.68% to 20.82% in U2932 cells and 40.20% to 26.67% in SUDHL2 cells (Fig. 3A and 3B). Moreover, we studied the mechanism of G_1_ phase arrest, and found the mRNA levels of *P21* was increased, and accompanied by *cyclin E* level was decreased (Fig. 3C). Taken together, these results indicate that BIX-01294 caused G_1_ phase arrest via increasing *P21* level and reducing *cyclin E* level and then inhibits proliferation in DLBCL cells.

### BIX-01294 induces apoptosis and activates apoptotic signaling pathway in human DLBCL cells

**Figure 4:**
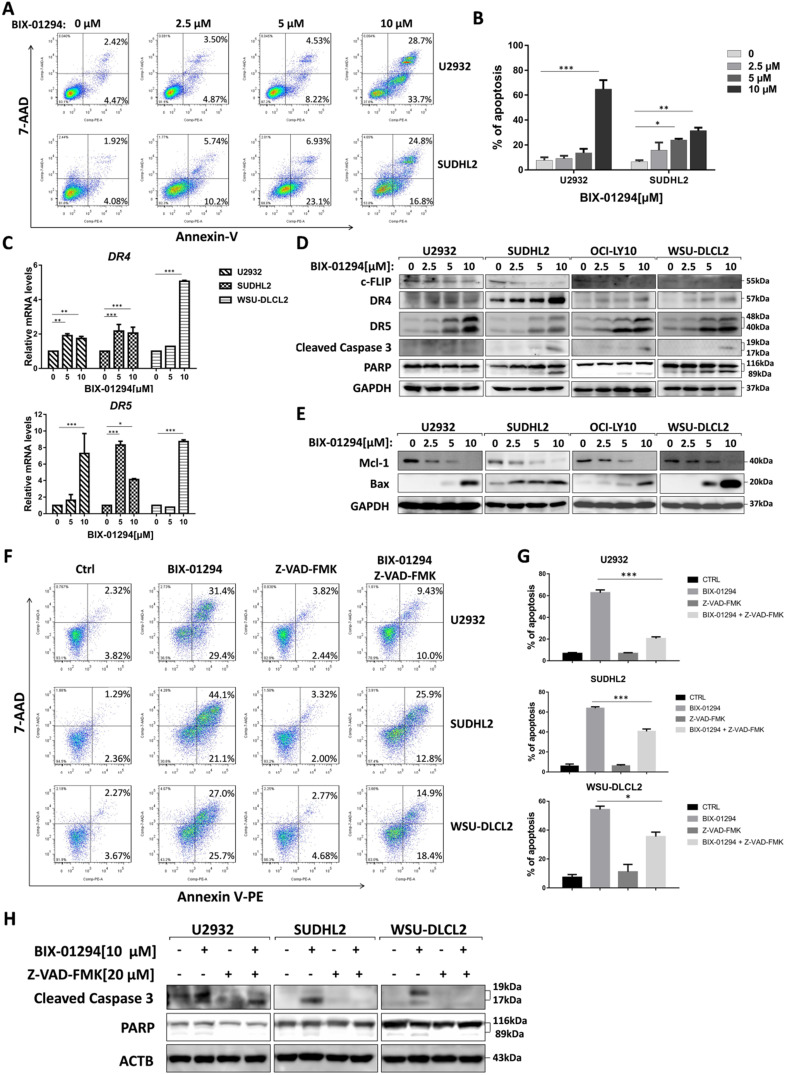
** BIX-01294 induces apoptosis and activates exogenous and endogenous apoptotic signaling pathway in human DLBCL cells. A,** U2932 and SUDHL2 cells were incubated with the indicated concentrations of BIX-01294 for 36 h, cells were harvested and subsequently stained with annexin-V-PE and 7-AAD and analyzed by flow cytometry for apoptosis. **B,** Percentages of apoptotic cells were determined from three independent experiments. **C,** U2932, SUDHL2, and WSU-DLCL2 cells were incubated with the indicated concentrations of BIX-01294 for 36 h. The expression of *DR4* and *DR5* mRNA was assessed by real-time PCR. **D and E,** U2932, SUDHL2, OCI-LY10, and WSU-DLCL2 cells were incubated with the indicated concentrations of BIX-01294 for 48 h. Then whole cells were harvested and subjected to western blot using c-FLIP, DR4, DR5, caspase 3, PARP (D) and Mcl-1, Bax (G) antibodies. **F-H,** U2932, SUDHL2, and WSU-DLCL2 cells were pretreated with Z-VAD-FMK for 1 h and then incubated with BIX-01294 for 48 h. Whole cells were harvested and subjected to analyze by flow cytometry for apoptosis (F) or western blot (H). Percentages of apoptotic cells were determined from three independent experiments (G). Error bars, mean ± SD. *P < 0.05; **P < 0.01; *** *P* < 0.001.

Furthermore, we performed flow cytometric assay to elucidate the apoptotic effect and found that BIX-01294 treatment induced U2932 and SUDHL2 apoptosis. As the concentration increases, the percentage of apoptosis was significantly increased (Fig. 4A and 4B). Particularly, 10 μM BIX-01294 induced about 62% of apoptotic cells in U2932. Meanwhile, we elucidated the apoptotic effect induced by BIX-01294 in Molt-4, Jurkat and PMBC cells as internal and external controls 21 (Supplementary Fig. 1). Then we examined whether the apoptotic signaling pathway was activated. First, we testified that mRNA levels of death receptors DR4 and DR5 were elevated in U2932, SUDHL2 and WSU-DLCL2 cells (Fig. 4C), suggesting BIX-01294 activated exogenous apoptosis pathway. By western blot, we also showed that the expression of anti-apoptotic protein c-FLIP was decreased, and the level of DR4 and DR5 was up-regulated in U2932, OCI-LY10, SUDHL2, WSU-DLCL2 cells (Fig. 4D). Conformably, BIX-01294 increased the cleaved forms of caspase 3 and PARP, further proved that BIX-01294 induces exogenous apoptosis pathway. Meanwhile, the experiments also showed that BIX-01294 down-regulated anti-apoptotic protein Mcl-1 expression and up-regulated pro-apoptotic protein Bax level (Fig. 4E), indicating BIX-01294 also activates endogenous apoptotic pathways. Pretreatment with the caspase inhibitor Z-VAD-FMK for 1 h decreased the cleaved forms of caspase 3 and PARP in U2932, SUDHL2, and WSU-DLCL2 cells (Fig. 4H). The percentage of BIX-01294 induced apoptosis was also significantly reduced with pretreatment with Z-VAD-FMK (Fig. 4F and 4G). Taken together, BIX-01294 activates exogenous and endogenous apoptotic signaling pathways in human DLBCL cells.

### BIX-01294 triggers autophagy in human DLBCL cells

**Figure 5:**
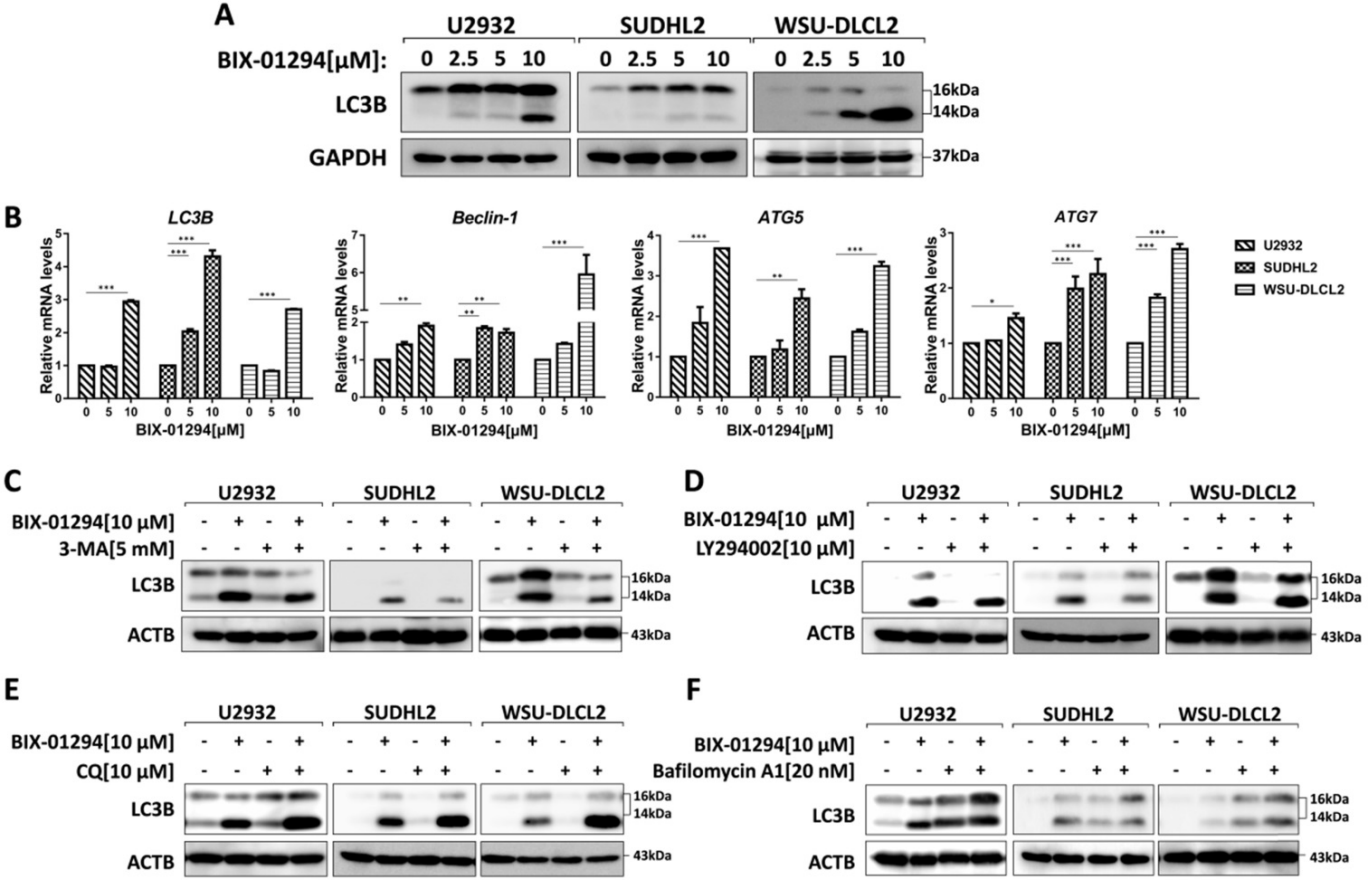
** BIX-01294 triggers autophagy in human DLBCL cells. A,** U2932, SUDHL2 and WSU-DLCL2 cells were incubated with the indicated concentrations of BIX-01294 for 48 h. Then whole cells were harvested and subjected to western blot analysis using LC3B antibody. **B,** U2932, SUDHL2, and WSU-DLCL2 cells were incubated with the indicated concentrations of BIX-01294 for 36 h. The expression of *LC3B, Beclin-1, ATG5* and* ATG7* mRNA was assessed by real-time PCR. Error bars, mean ± SD. *P < 0.05; **P < 0.01; *** *P* < 0.001. **C- F,** U2932, SUDHL2, and WSU-DLCL2 cells were pretreated with 3-MA (5 mM) (C), LY294002 (10 µM) (D), chloroquine (CQ, 10 µM) (E) or bafilomycin A1 (20 nM) (F) for 1 h and then incubated with BIX-01294 for 48 h. Afterward, the whole cells were harvested and subjected to western blot analysis using the LC3B antibody.

To determine whether BIX-01294 induced autophagy, we treated three human DLBCL cell lines with BIX-01294. LC3B is the hallmark of autophagy, so we examined the expression of LC3B using western blot and qPCR. After treatment with BIX-01294 at the indicated concentration for 48 h, both protein and mRNA levels of LC3B increased in DLBCL cells (Fig. 5A and 5B). We also examined other autophagy markers such as Beclin-1, ATG5, and ATG7, and their mRNA levels were elevated in our tested cells (Fig. 5B). Furthermore, we co-treated DLBCL cells with BIX-01294 and autophagy inhibitors. 3-MA or LY294002, both block the upstream steps of autophagy, reduced LC3B expression when combined with BIX-01294 (Figs. 5C and 5D). While the combination of BIX-01294 and chloroquine (CQ) or bafilomycin A1, both block the downstream steps of autophagy, increased the expression of LC3B (Fig. 5E and 5F). Collectively, these results confirm that BIX-01294 triggers autophagy in human DLBCL cells.

### BIX-01294 activates ER stress

**Figure 6:**
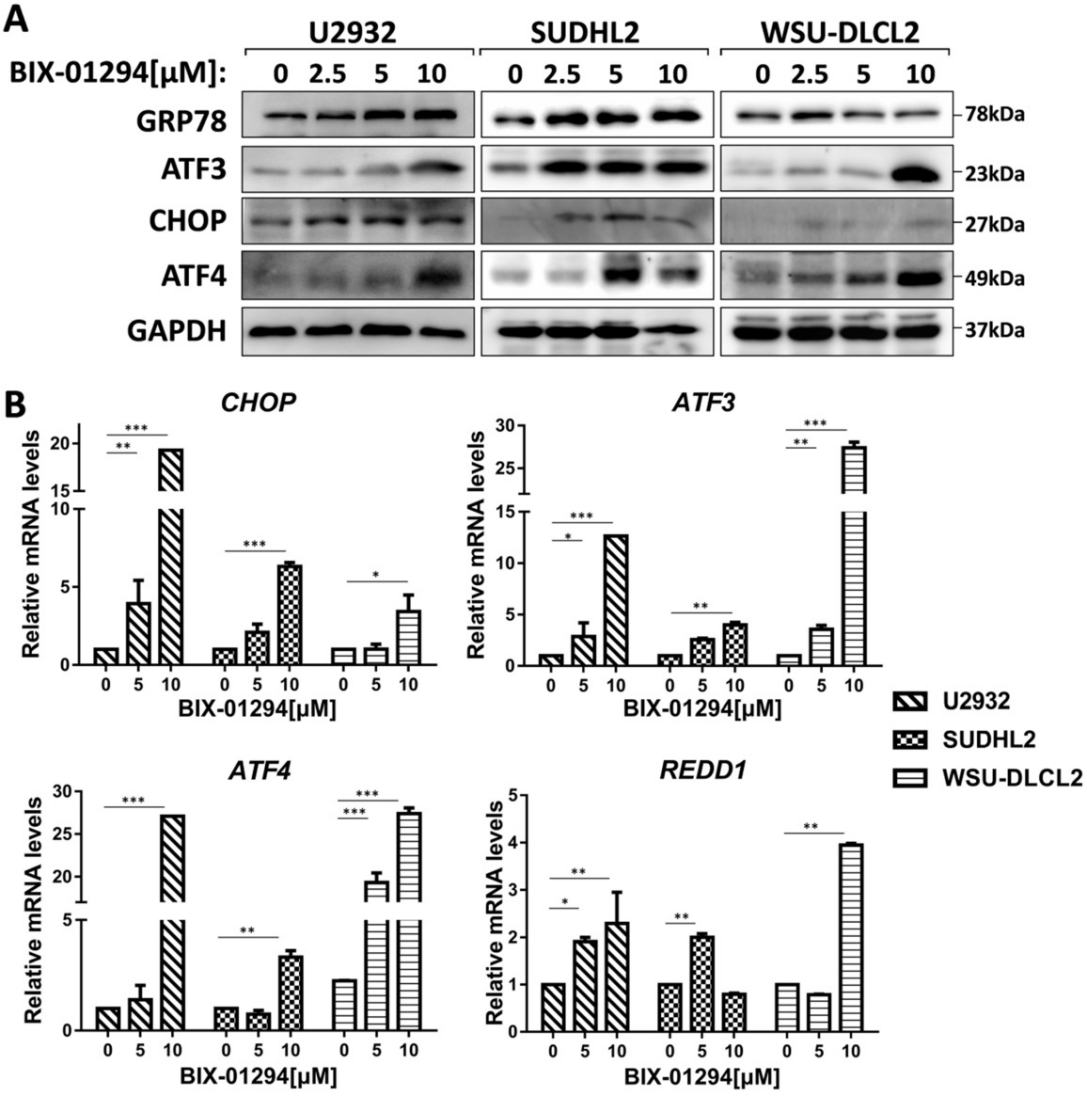
** BIX-01294 activates ER stress. A,** U2932, SUDHL2, and WSU-DLCL2 cells were incubated with the indicated concentrations of BIX-01294 for 48 h. Then whole cells were harvested and subjected to western blot analysis using GRP78, ATF3, CHOP, and ATF4 antibodies. **B,** U2932, SUDHL2, and WSU-DLCL2 cells were incubated with the indicated concentrations of BIX-01294 for 36 h. The expression of *ATF3*, *CHOP*, *ATF4* and *REDD1* mRNA was assessed by real-time PCR. Error bars, mean ± SD. *P < 0.05; **P < 0.01; ***P < 0.001.

To elucidate the mechanism of BIX-01294-induced apoptosis and autophagy in DLBCL cells, we studied the active stage of endoplasmic reticulum (ER) stress. We examined the expression of GRP78, CHOP, ATF3, and ATF4, which are regarded as important protein markers of ER stress and found that their expression was enhanced when treated with BIX-01294 (Fig. 6A). At the same time, we found that the mRNA levels of *CHOP, ATF3, ATF4,* and *REDD1* were markedly increased (Fig. 6B). Collectively, these results indicate BIX-01294 activates ER stress.

### ATF3 expression is required for BIX-01294-induced death

**Figure 7:**
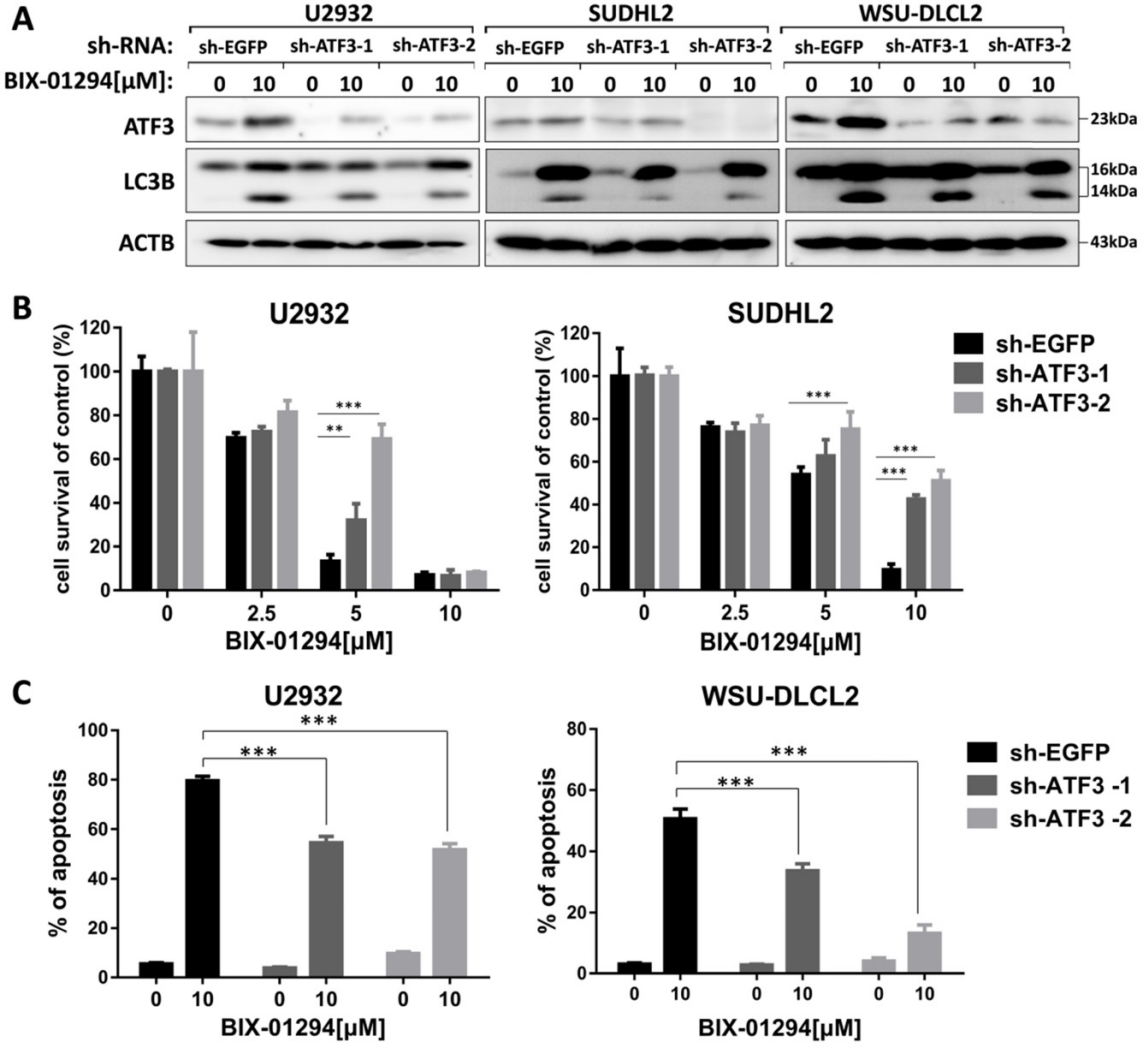
** ATF3 expression is required for BIX-01294-induced apoptosis and autophagy.** U2932, SUDHL2, and WSU-DLCL2 cells were transduced with ATF3-shRNA and control-shRNA lentivirus and constructed stable knockdown cell lines. Then cells were incubated with BIX-01294 for 48 h. **A,** Whole cells were harvested and subjected to western blot analysis using ATF3 and LC3B antibodies. **B,** CCK-8 assay was performed to detect the viability. **C,** The apoptotic rates were detected with Annexin V/7-AAD by flow cytometry, and the percentages of cell apoptosis was determined from three independent experiments. Error bars, mean ± SD. ** *P* < 0.01;*** *P* < 0.001.

As ATF3 expression is upregulated in BIX-01294 induced cell death, we wondered whether ATF3 influenced apoptosis and autophagy induced by BIX-01294. We performed the shRNA knockdown assay to inhibit ATF3 expression (Fig. 7A) and found that inhibition of ATF3 upregulation decreased the expression of LC3B, indicating ATF3 contributed to BIX-01294 induced autophagy. CCK-8 assay showed that the viability was increased in ATF3 abrogated-U2932 and SUDHL2 cells after exposure to BIX-01294 (Fig. 7B). Consistently, the percentage of apoptosis was significantly decreased in ATF3 knockdown cells than that in the control cells by Annexin V/7-AAD (Fig. 7C). Taken together, we conclude that ATF3 expression is required for BIX-01294-induced autophagy and apoptosis.

### Inhibition of ATF4 expression decreases BIX-01294-induced death via ATF3

**Figure 8:**
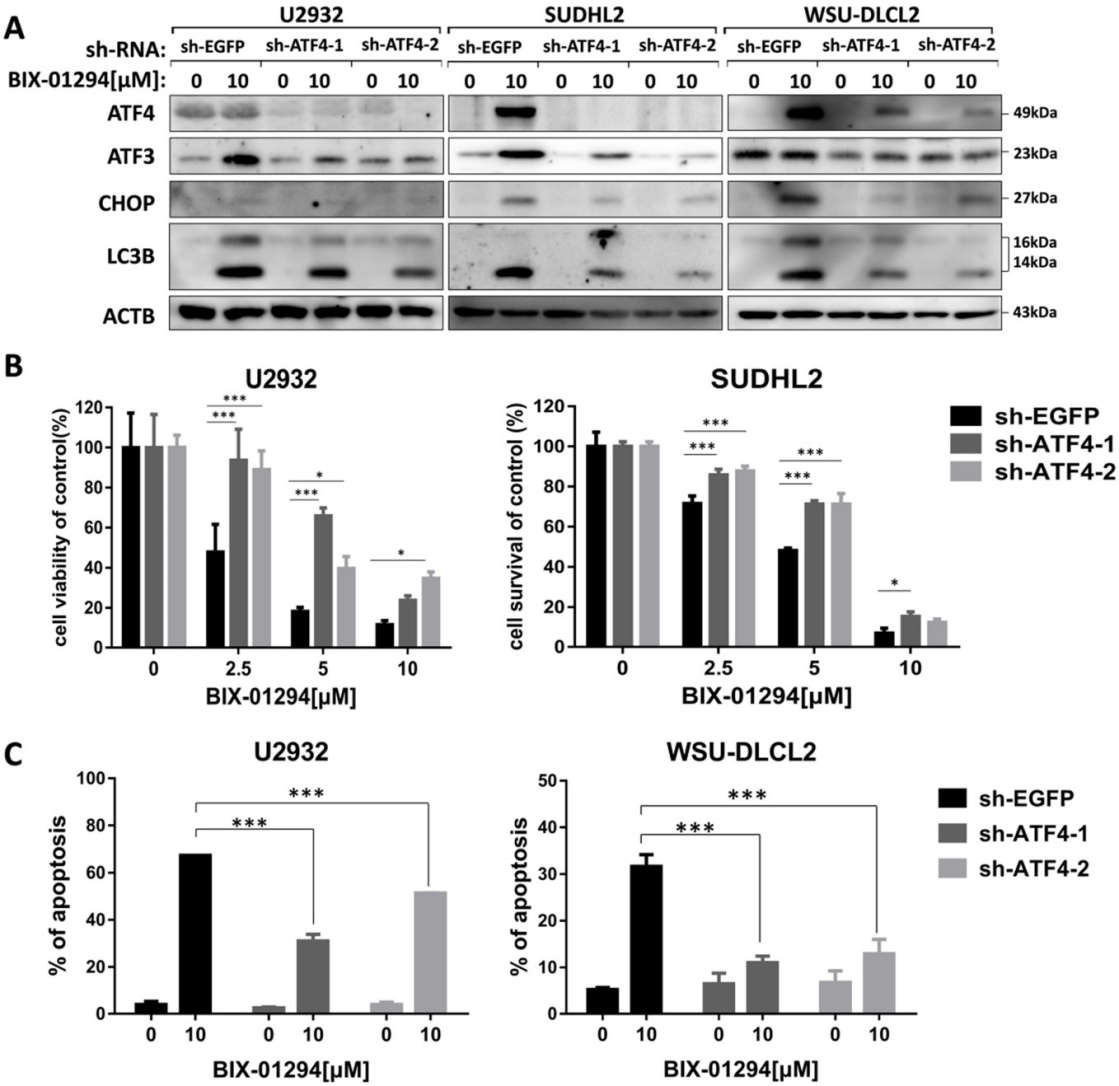
** Inhibition of ATF4 expression decreases BIX-01294-induced death via ATF3.** U2932, SUDHL2, and WSU-DLCL2 cells were transduced with ATF4-shRNA and control-shRNA lentivirus and constructed stable knockdown cell lines. Then cells were incubated with BIX-01294 for 48 h. **A,** Whole cells were harvested and subjected to Western blot analysis using ATF4, CHOP, ATF3, and LC3B antibody. **B,** CCK-8 assay was performed to detect the viability. **C,** The apoptotic rates were detected with Annexin V/7-AAD by flow cytometry, and the percentages of cell apoptosis were determined from three independent experiments. Error bars, mean ± SD. * *P* < 0.05; *** *P* < 0.001.

We then explored whether BIX-01294-induced ATF4 upregulation would contribute to LC3B expression. To do this, we successfully blocked BIX-01294-induced ATF4 expression using ATF4 shRNA in U2932, SUDHL2, and WSU-DLCL2 cells (Fig. 8A). We found that the inhibition of ATF4 expression decreased the expression of LC3B, indicating ATF4 expression contributed to autophagy. Besides, we noticed that after abolishing ATF4 expression, BIX-01294-induced cell survival inhibition was decreased in U2932 and SUDHL2 cells as determined using CCK-8 assay (Fig. 8B). Accordingly, the percentage of apoptotic cells was reduced in ATF4-shRNA transfected U2932 and WSU-DLCL2 cells than that in control cells (Fig. 8C). We also showed that suppressed ATF4 expression inhibited ATF3 and CHOP expression in DLBCL cells (Fig. 8A). Taken together, we speculate that BIX-01294 treatment induces ATF4 upregulation, and then promotes ATF3 and CHOP expression, subsequently contributing to BIX-01294-mediated autophagy and apoptosis.

## Discussion

Overexpression of EHMT2 may be associated with a poor prognosis in patients with various types of malignant cancers including lung cancer, ovarian cancer, esophageal squamous cell carcinoma, and so on 14-16. Several EHMT2 inhibitors have demonstrated good anti-tumor activity by preventing cancer cell proliferation and inducing apoptosis in many types of tumors, such as T lymphoblastic leukemia cells, bladder cancer, glioma tumor, renal cancer, and so on 10, 19-21. However, the role of BIX-01294 and the involvement of EHMT2 in DLBCL are not well studied to date. In this study, we showed that with the increased concentration of BIX-01294, the cell survival rate of DLBCL was inhibited, and the cell cycle was arrested in G_1_ phase. Moreover, BIX-01294 induced DLBCL cells apoptosis, and both endogenous and exogenous apoptotic pathways were activated. Furthermore, we found activating the ER stress pathway is one of the underlying mechanisms of how BIX-01294 triggers apoptosis.

In the present study, we showed that BIX-01294 up-regulated the expression of death receptors DR4 and DR5 along with down-regulating anti-apoptotic protein c-FLIP expression, indicating that BIX-01294 induced exogenous apoptosis pathway. Meanwhile, anti-apoptotic protein Mcl-1 expression is decreased, and pro-apoptotic protein Bax expression is increased, suggesting that BIX-01294 also activated endogenous apoptotic pathways. These results were consistent with the results of Woo et al. that demonstrated BIX-01294 induced DR5 expression in renal cancer 19, and the results of Huang et al. that showed BIX-01294 increased Bax expression in leukemia cells 21.

Studies have shown that severe long-term ER stress could induce apoptosis, and DR5 expression is regulated by CHOP and ER stress 22, 23. We observed that BIX-01294 elevated CHOP expression in protein and mRNA levels. Other important components of ER stress, including GRP78, ATF3, ATF4, and REDD1, were all increased when human DLBCL cells were treated with BIX-01294. These results implied BIX-01294 activated ER stress, which is consistent with other studies that BIX-01294 triggers ER stress response in bladder cancer cells 10. Next, we analyzed the role of ATF3 and ATF4 in BIX-01294 induced apoptosis. We found that abolishing ATF3 expression effectively decreased the apoptosis induced by BIX-01294. Silencing ATF4 expression also suppressed BIX-01294-mediated cell proliferation inhibition and cell apoptosis. Meanwhile, we detected that inhibiting ATF4 expression obviously decreased ATF3 expression. So these results suggesting that ATF4 facilitated apoptosis through regulating ATF3, and both ATF3 and ATF4 are required for BIX-01294 induced apoptosis. Studies reported that BIX-01294 up-regulated ATF4/CHOP-dependent DR5 expression 24, however, whether ATF4 expression contributes to BIX-01294-induced CHOP and DR5 expression in DLBCL cells still needs to be investigated.

Previous studies reported that BIX-01294 induced autophagy by increasing Beclin-1 expression in breast cancer and human promyelocytic leukemia 13, 25. Consistently, we proved that BIX-01294 improved LC3B expression both at protein and mRNA levels, and the mRNA levels of autophagy-related molecules *Beclin-1*, *ATG5*, and *ATG7* were also elevated. In addition, we examined whether BIX-01294 could induce the autophagic flux in DLBCL cells. Combination of BIX-01294 and autophagy inhibitors, such as upstream steps of autophagy inhibitor, 3-MA, or LY294002 decreased LC3B-II formation. In contrast, co-treatment BIX-01294 with downstream steps of autophagy inhibitor, CQ, or bafilomycin A1 increased LC3B-II expression. These results indicated that BIX-01294 induces DLBCL cells autophagy. In-depth studies discovered that inhibition of ATF3 or ATF4 resulted in the decrement of LC3B expression, suggesting that ER stress is involved in regulating autophagy. This is consistent with other studies that ATF4 regulates autophagy 26, 27. Studies have shown that ATF3 facilitates chemical compounds induced autophagy-associated cell death 28, while other studies have shown that ATF3 negatively regulates cellular autophagy 29. Our study supported that ATF3 is conducive to autophagy.

Autophagy is also known as type II programmed cell death that is involved in the development of various diseases 30, 31. It can also be activated by multiple stress stimuli, so the interaction regulation between autophagy and apoptosis is complex. In some cases, autophagy is the cell survival pathway and inhibits apoptosis; while in other cases, autophagy itself also induces cell death, cooperates with apoptosis 32-34. The two pathways are related and regulate each other. So a comprehensive and in-depth study on the interaction between autophagy and apoptosis may lead to a breakthrough in the understanding and treatment of diseases such as tumors 35, 36. In our study, we showed that BIX-01294 induced apoptosis and autophagy simultaneously, and ER stress regulated the two cellular physiological processes, so the specific mechanism still needs to be further studied.

In conclusion, we demonstrated that BIX-01294 inhibits the expression of EHMT2 in human DLBCL cells, and then inhibits cell proliferation by inducing G_1_ phase arrest and inducing apoptosis through both endogenous and exogenous apoptotic pathways. In addition, BIX-01294 triggers autophagy, and activates ER stress in human DLBCL cells. Both key components of ER stress, ATF3, and ATF4, are required for BIX-01294-induced apoptosis and autophagy.

## Supplementary Material

Supplementary figure S1.

## Figures and Tables

**Table 1:** Sequences of primers for RT-qPCR

Name	Sequences
EHMT2	5′-AGTGTGACCCTGACCTCTGT--3′ (forward)
	5′-AGATGGTGCCAGCAATAGAT-3′ (reverse)
P21	5′-TGAGCCGCGACTGTGATG-3′ (forward)
	5′-GTCTCGGTGACAAAGTCGAAGTT-3′ (reverse)
CyclinE	5′-TTCTTGAGCAACACCCTCTTCTGCAGCC-3′ (forward)
	5′-TCGCCATATACCGGTCAAAGAAATCTTGTGCC-3′ (reverse)
DR4	5′-TCCAGCAAATGGTGCTGAC-3′ (forward)
	5′-GAGTCAAAGGGCACGATGTT-3′ (reverse)
DR5	5′-CCAGCAAATGAAGGTGATCC-3′ (forward)
	5′-GCACCAAGTCTGCAAAGTCA-3′ (reverse)
ATF4	5′-GCTAAGGCGGGCTCCTCCGA-3′ (forward)
	5′-ACCCAACAGGGCATCCAAGTCG-3′ (reverse)
ATF3	5′-TGATGCTTCAACACCCAGGCC-3′ (forward)
	5′-AGGGGACGATGGCAGAAGCA-3′ (reverse)
CHOP	5′-CATCACCACACCTGAAAGCA-3′ (forward)
	5′-TCAGCTGCCATCTCTGCA-3′ (reverse)
REDD1	5′-ACGCACTTGTCTTAGCAGTT-3′ (forward)
	5′-TAAGCCGTGTCTTCCTCC-3′ (reverse)
LC3B	5′-CGCACCTTCGAACAAAGAG-3′ (forward)
	5′-CTCACCCTTGTATCGTTCTATTATCA-3′ (reverse)
Beclin1	5′-GGTTGAGAAAGGCGAGACAC-3′ (forward)
	5′-GATGGAATAGGAGCCGCCAC-3′ (reverse)
ATG7	5′-ACCCAGAAGAAGCTGAACGA-3′ (forward)
	5′-CTCATTTGCTGCTTGTTCCA-3′ (reverse)
ATG5	5′-TTTGAATATGAAGGCACACC-3′ (forward)
	5′-TGCAATCCCATCCAGAGTTG-3′ (reverse)
β-Actin	5′-CTCCATCCTGGCCTCGCTGT-3′ (forward)
	5′-GCTGTCACCTTCACCGTTCC-3′ (reverse)

## Abbreviations

ABC: activated B-cell-like
ATF: activating transcription factor
CCK-8: cell counting kit-8
CHOP: C/EBP homologous protein
DR: death receptor
DLBCL: diffuse large B-cell lymphoma
EFS: event-free survival
EHMT2: euchromatic histone lysine methyltransferase 2
EMT: epithelial-mesenchymal transition
ER: endoplasmic reticulum
GCB: germinal center B cell-like
NHL: non-Hodgkin lymphoma
H3K9: histone H3 lysine 9
R-CHOP: rituximab, cyclophosphamide, doxorubicin, vincristine and prednisone
RT-qPCR: real-time quantitative polymerase chain reaction
PBMC: peripheral blood mononuclear cells
SD: standard deviation
OS: overall survival
