# Supplementary material for: EHMT2 inhibitor BIX-01294 induces endoplasmic reticulum stress mediated apoptosis and autophagy in diffuse large B-cell lymphoma cells
Source: J Cancer. 2021 Jan 1;12(4):1011–22. doi: 10.7150/jca.48310 (PMC7797660; doi:10.7150/jca.48310)

Supplementary material

Figure 1. BIX-01294 induces apoptosis in human Molt-4 and Jurkat cells

A. Molt-4 and Jurkat cells were incubated with the indicated concentrations of BIX-01294 for 36 h, cells were harvested and subsequently stained with annexin-V-PE and 7-AAD and analyzed by flow cytometry for apoptosis.

B. Human PBMC were incubated with the indicated concentrations of BIX-01294 for 36 h, cells were harvested and subsequently stained with annexin-V-PE and 7-AAD and analyzed by flow cytometry for apoptosis.

C. Molt-4 and Jurkat cells were incubated with the indicated concentrations of BIX-01294 for 48 h. Then whole cells were harvested and subjected to western blot using caspase 8, caspase 3, and PARP antibodies.

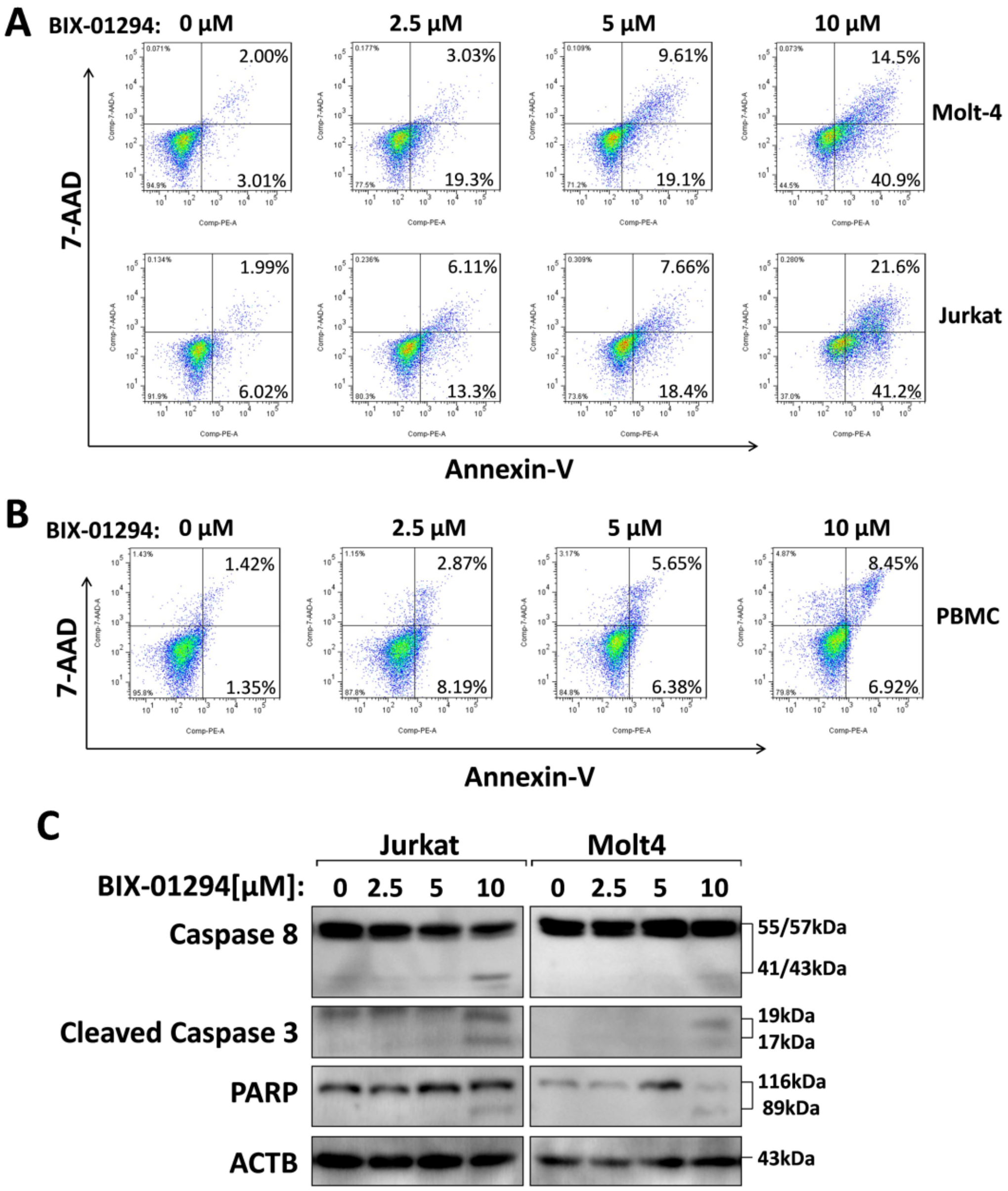

Supplement: Supplementary file 1 — Supplementary figure S1. [file jcav12p1011s1.pdf]
